# The lived complexity of phantom limb telescoping: insights from a qualitative study

**DOI:** 10.3389/fresc.2026.1747066

**Published:** 2026-07-21

**Authors:** Andrea Aternali, Heather Lumsden-Ruegg, Nicole Dimitrova, Lora Appel, Sander L. Hitzig, Amanda L. Mayo, Joel Katz

**Affiliations:** 1 Department of Psychology, York University, Toronto, Ontario, Canada; 2 School of Health Policy & Management, York University, Toronto, Ontario, Canada; 3 St. John’s Rehab Research Program, Sunnybrook Research Institute, Sunnybrook Health Sciences Centre, Toronto, Ontario, Canada; 4 Department of Occupational Science and Occupational Therapy, Temerty Faculty of Medicine, University of Toronto, Toronto, Ontario, Canada; 5 Rehabilitation Sciences Institute, Temerty Faculty of Medicine, University of Toronto, Toronto, Ontario, Canada; 6 Dalla Lana School of Public Health, University of Toronto, Toronto, Ontario, Canada; 7 Department of Medicine, Temerty Faculty of Medicine, University of Toronto, Toronto, Ontario, Canada

**Keywords:** amputation, lived experience, phantom limb, qualitative research, telescoping

## Abstract

Phantom limb telescoping, the perceived shortening of a missing limb, remains an underexplored phenomenon despite its relatively frequent occurrence after limb loss. Existing literature has focused on quantitative or neurophysiological approaches, with limited attention to lived experience. This study examined first-person accounts of telescoping among 12 participants with limb loss (11 men, one woman; mean age 50.1 years; mean 3.9 years post-amputation) using semi-structured interviews. The aim was to explore the lived experience of phantom limb telescoping among people who have undergone limb amputation, including their interpretations of telescoping, the emotional responses it evoked, and its impact on post-amputation adaptation. Data were analyzed using an inductive thematic analysis within a constructionist framework. Four themes were identified: *(1) Heterogeneity in the experience of telescoping, (2) Telescoping as unusual but not distressing, (3) Social and emotional consequences of living with telescoping,* and *(4) Adaptation to an altered phantom limb.* Participants described telescoping as unusual but not distressing. Participants described feeling more concerned by factors related to post-amputation adjustment or phantom limb pain than the sensation of having a shorter phantom limb. Participants reported rarely discussing telescoping with social supports or healthcare providers. Their experience of telescoping varied in onset and presentation and were shaped by psychological and social factors. These findings support the suggestion that patients may benefit from education on telescoping soon after amputation and open discussions with healthcare providers to normalize telescoping and reduce feelings of uncertainty surrounding this sensation. Integrating patient-centered qualitative insights into rehabilitation may better address the full spectrum of post-amputation experiences.

## 1 Introduction

Limb loss, whether due to trauma, disease, or infection, often gives rise to a range of phantom sensations (1). The most common of these include pain, tingling, cramping, temperature changes, pressure, and a sense of movement, all referred to the missing, or phantom limb (2). Many phantom sensations, especially phantom limb pain (PLP), have been extensively documented in the clinical and psychological literatures (3). However, one of the more unique or unusual post-amputation phenomena to occur, known as phantom limb telescoping, remains relatively underexplored. Telescoping refers to the sensation in which the phantom hand or foot is perceived to gradually move closer to the residual limb. The phantom extremity may eventually even retract completely into the residual limb where it is also perceived to be much smaller (e.g., shrinking) in proportion to the original size of the hand or foot (2, 4). Although telescoping is estimated to occur in approximately 14%–32% of individuals with limb loss (5–8), with variability likely reflecting differences in measurement approaches (e.g., lifetime vs. point vs. period prevalence), it remains an underexplored sequela of amputation. The lack of attention to telescoping in the limb loss literature may reflect its frequent overshadowing by more frequently reported and distressing phenomena, like PLP (9). Telescoping reflects a complex interplay of sensory, cognitive, and emotional processes (10). Its presence profoundly alters an individual's perception of their phantom limb and represents one of many unfamiliar sensations that individuals experience following amputation. Telescoping may also be associated with PLP, where some research has suggested that telescoping is associated with the presence of PLP (11, 12) or increased PLP intensity (6, 13). Other research has found that telescoping is associated with the absence of PLP (14, 15). While this evidence is mixed and in need of further investigation, it highlights the potential clinical relevance of telescoping, which is a phenomenon that relatively little is known about in terms of how individuals experience and make sense of it. Understanding this may clarify variability in post-amputation experiences, as well as provide insights on its impact on quality of life and post-amputation adaptation.

There remains a relative dearth of literature qualitatively exploring the lived experience of telescoping. Bjorkman, Arner (16) employed qualitative interviews to better understand the experiences of individuals with PLP (*N* = 28), and found that while some participants described telescoping sensations, these were incidental findings secondary to pain. Similarly, Trevelyan, Turner (17) investigated the impact of PLP on quality of life, noting instances of telescoping among participants, yet without examining the psychosocial impact of telescoping. Early work by Frank and Lorenzoni (18) considered the influence of waking vs. sleeping states on the experience of telescoping in individuals with both full-length and shortened phantom limbs. However, their study did not address how these variations shaped the individual's subjective or emotional experience, leaving the affective dimensions of telescoping underexplored.

More recently, Middleton and Ortiz-Catalan (19) examined telescoping in the context of neuromusculoskeletal prosthesis use, and observed that all three participants included in their study experienced phantom limb shortening in the absence of their prosthesis and lengthening to align with the seen position of the prosthetic hand when it was worn. Yet, the study did not delve into the psychological or emotional implications of this phenomenon. Collectively, these gaps highlight the need for further qualitative research centered on the lived experiences of individuals with limb loss, particularly regarding how telescoping influences their daily lives, mental health, and adaptation to limb loss.

To address these gaps, the present study aims to better understand the post-amputation experience via first-person accounts of phantom limb telescoping. The primary research question was: What is the lived experience of phantom limb telescoping among people who have undergone limb amputation? In particular, the study sought to explore how participants interpreted telescoping, the emotional responses it evoked, and its impact on post-amputation adaptation. This approach aimed to capture the complexity and individuality of post-amputation experiences, which are often difficult to capture through quantitative methods alone. By attending to phantom limb phenomena that are often overlooked, this study may help align clinical and research agendas more closely with the needs, challenges, and priorities of individuals living with limb loss.

## 2 Methods

### 2.1 Study design

This study used a qualitative research design and inductive thematic analysis (20). The study is part of a broader research project exploring the relationship between telescoping and PLP. It was approved by the Research Ethics Boards of Sunnybrook Health Sciences Centre (REB #3071) and York University (HPRC #e2019-138). The participants provided written and verbal informed consent to participate in this study. The study, methods, and description of one participant's experience have also been published elsewhere (21, 22). The Consolidated Criteria for Reporting Qualitative Studies guidelines for reporting qualitative research was followed (23), and the Joanna Briggs Institute (JBI) critical appraisal checklist for qualitative research was followed to minimize risk of bias (24).

### 2.2 Reflexivity statement

The first author (A.A.) conducted the semi-structured interviews and data analysis following graduate-level training in qualitative methods. She had no prior relationship with any of the participants and did not provide clinical care to them. She limited reporting any characteristics about herself (e.g., biases, assumptions, reasons for exploring the research topic) to participants. Participants were informed prior to their participation that the study aimed to better understand factors that contribute to post-amputation pain.

### 2.3 Participants and data collection

Individuals who had undergone limb amputation were recruited from June 1, 2019 to December 16, 2024 through advertisements posted at St. John's Rehab (Sunnybrook Health Sciences Centre), a rehabilitation centre in Toronto, Canada, and through social media posts shared by the authors and limb loss organizations (e.g., Amputee Coalition of Toronto). Interested individuals were asked to contact the study coordinator (AA), who screened for eligibility. Individuals were included if they were at least 18 years old with the ability to read and speak English fluently. They also were required to have had an upper and/or lower limb amputated more than three months prior to recruitment, to ensure participants were beyond the acute post-operative phase. Participants who were cognitively impaired, diagnosed with severe psychopathology (e.g., active psychotic disorder, dementia, etc.), or had significant issues in visual impairment were not eligible to participate in the study. All participants were first given a link to an online consent form, where they provided written informed consent.

Once consent was obtained, participants were then asked to complete a series of patient-reported outcome measures via online questionnaires. If participants endorsed telescoping when filling out the questionnaires, they were invited to an optional interview, which took place either in person (prior to the COVID-19 pandemic) at a rehabilitation hospital (St. John's Rehab) or virtually via secure videoconferencing. The interviewer used a semi-structured interview guide, consisting of a series of open-ended questions and probes to elicit a deeper understanding of the participant's perceptions and experiences with their phantom limb. Individual interviews were estimated to take approximately 45 min.

After several initial interviews, the first author (A.A.) consulted with a senior member of the team trained in qualitative methods (S.L.H.) to review the semi-structured interview guide. This review confirmed that the initial questions were appropriately capturing relevant data and revisions to the guide were not required, serving to confirm the guide's effectiveness and alignment with the study objectives. To minimize the risk of error and investigator recall bias, the interviewer took notes during the interview when the participant responded to specific experiential questions. All interviews were digitally recorded and later transcribed verbatim. H.L.R. took a leading role and transcribed the majority of the interviews, while N.D. transcribed several and assisted A.A. during the analysis phase. Transcripts were not returned to participants for comment and/or correction, nor were participants consulted for feedback on the findings. Data saturation was determined to have occurred when new information (e.g., new codes) related to the research question was not forthcoming (25).

### 2.4 Measures

Participants responded to questionnaires evaluating demographic (e.g., month and year of birth), clinical (e.g., month and year of amputation), psychosocial (e.g., Connor-Davidson Resilience Scale-2) and pain-related characteristics. Participants rated their PLP and Residual limb pain (RLP) using a 0–10 Numeric Rating Scale, where 0 indicated no pain and 10 indicated the worst pain imaginable. Participants were asked whether they experienced PLP and RLP several times a week or more, over the past three months. A detailed description of all measures are reported elsewhere (22).

The semi-structured interviews began with the investigator obtaining verbal consent. The interviewer then introduced the purpose of the study and invited the participants to share a description of their phantom limb(s). Participants were asked to describe their phantom limb and any associated pain, including onset, duration, quality, and changes over time. RLP and phantom limb shrinking (i.e., the feeling as though their phantom hand or foot was smaller in size) were similarly examined. The open-ended interview questions allowed participants to describe a variety of phantom limb experiences. While all painful and non-painful phantom sensations were explored, those findings were not reported because they were not the focus of the present study, only described by one participant, and/or not discussed in sufficient detail. A substantial portion of the interview focused on phantom limb telescoping, including when it was first noticed, how it was experienced, and how it may have changed in relation to emotional states, prosthesis use, or other contextual factors. Participants were also asked how various post-amputation sensations, such as telescoping, impacted their physical health, mental wellbeing, and relationships. The interview concluded with an open-ended invitation for participants to share any further reflections on their phantom limb experience. The semi-structured interview guide is listed in Table 1. The interview guide was developed based on clinical experience and existing literature on PLP (17), as no prior qualitative research has specifically investigated the lived experience of telescoping.

**Table 1:** The list of interview questions.

No.	QUESTIONS
1.	How would you describe your phantom limb, and any pain associated with it?
	When have you experienced it? Has it only been in the past or also presently? When did it first start? Had it ever gone away and then came back? How would you describe the type of pain, if any? Has the feeling changed over time?
2.	How would you describe your residual limb pain, if any, which is the pain that you feel in the part of your remaining limb after your amputation?
	When have you experienced it? Has it only been in the past or also presently? When did it first start? Had it ever gone away and then came back? How would you describe the type of pain, if any? Has the feeling changed over time?
3.	Have you experienced phantom limb "telescoping"?
	When did it first happen? What were your initial reactions? How have others reacted when you have tried to explain it? What have been your experiences with it? Is it a constant length?
4.	Have you experienced any shrinkage of the phantom limb?
	When did it first happen? What were your initial reactions? What have been your experiences with it? Is it a constant size? How has it influenced areas of your life negatively or positively?
5.	When thinking about the different types of sensations associated with your amputation (e.g., phantom limb, telescoping, residual limb, etc.), how have any of these affected your day to day life?
	Impact on ability to maintain your physical health? Impact on your mental wellbeing?&&&&&&Impact on your relationships with others?
6.	Is there anything else about your phantom limb that you would like to discuss that we did not touch upon?

### 2.5 Analysis

Data collection and analyses were carried out through an iterative and reflexive process, allowing the data to inform ongoing analytic decisions. The inductive thematic analysis approach was used to ensure that themes were identified from the participants' reported experiences rather than imposing pre-existing frameworks (20). A constructionist epistemological perspective was adopted to reflect the assumption that experiences of telescoping are shaped by psychological, social, and emotional contexts (26). Consistent with this perspective, the analysis was primarily reflexive and interpretive in nature, emphasizing researcher engagement with participants' accounts and the co-construction of meaning. At the same time, limited involvement of a second analyst was incorporated to encourage reflexive dialogue, challenge assumptions, and enhance the credibility of the analytic process. As such, the approach reflects a combination of reflexive thematic analysis and collaborative coding practices, with the goal of deepening interpretation.

To support the organization and interpretation of the qualitative data, NVivo 15, a qualitative data analysis software, was used for managing, coding, and retrieving interview transcripts.

All transcripts were read in full by the primary analyst (A.A.) to refamiliarize herself with the data. She completed initial coding on a line-by-line basis, with codes identified directly from participants' accounts. While the analysis was inductive, the researcher's prior knowledge of chronic pain and phantom limb sensations informed her interpretive lens during analysis. Codes were reviewed, refined, and grouped as analysis progressed. To ensure the consistency in code application, a second researcher (N.D.) independently coded three transcripts. Code comparisons were used to explore differences in interpretation and refine code definitions, rather than to achieve statistical agreement. This approach is guided by qualitative researchers who have argued that limiting double-coding can enhance reflexivity and analytic depth, while an over-reliance on inter-coder agreement may encourage more superficial coding frameworks (27). The researchers met to compare code application and reconciled discrepancies through discussion.

Subsequently, codes were compared across transcripts to identify patterns, similarities, and differences within the dataset. Related codes were then grouped into categories based on their underlying meaning. Exemplary quotations that were able to clarify exemplify the key features of each code were carefully selected form the dataset. This helped ensure that the analysis was grounded in participants' narratives. Analytic notes were developed for each category to define its scope and significance. Reflexive memos were also used throughout the analysis to track how the researcher's perspective might have influenced coding and grouping.

Themes and subthemes were developed by organizing these groups into higher-order patterns that captured shared meanings across participants. Themes were refined through feedback from the larger research team. This process involved reviewing and reorganizing themes to ensure that they accurately represented the data. Throughout the data analysis process, the team engaged in reflexive dialogue, critically examining their assumptions and actively exploring alternative interpretations. Hence, investigator triangulation was used throughout the analysis process, and this commitment to reflexivity and methodological rigor contributed to the credibility and overall quality of the qualitative analysis (28).

## 3 Results

Twelve individuals who experienced telescoping participated in this study, including 11 men and one woman. No participants who expressed interest subsequently declined or withdrew from the study. Participants had a mean age of 50.1 years (SD = 12.2; range = 35–67 years). All participants had one limb amputated with the exception of one participant (P012) who had undergone quadruple limb amputations. The average time since amputation was 3.9 years (SD = 3.5; range = 0.5–12 years). On average, interviews lasted 47.1 min (SD = 13.7; range = 26.2–78.2 min). Only the investigator and participant were present during each interview. The sample consisted primarily of participants with trauma- or cancer-related limb loss (*n* = 7). Eight of 12 participants (67%) reported moderate to severe PLP in the past week. Seven participants also reported moderate to severe RLP in the past week. The mean PLP and RLP scores were 4.6 (SD = 3.6) and 4.9 (SD = 3.4), respectively. For the participant with four limb amputations (P012), PLP and RLP ratings were averaged across both upper limbs as those were the limbs in which he experienced telescoping. An average was used to generate a single pain score for this participant to allow for easy comparison of pain ratings reported for participants with a single limb amputation. This participant reported that he did not experience PLP in either upper limb. Additional demographic and amputation-related details, including side, level, and etiology, are presented in Table 2.

**Table 2:** Participant demographics (*N* = 12).

Id	Age	Sex	Ethnicity	Highest degree of education	Marital status	Living with	Date(s) of interview	Interview duration (min: sec)	# limbs amputated	Time since amputation (years)	Etiology	Extremity/level	Side	RLP (NRS)	PLP (NRS)
001	59	Male	White	Professional	Married	Spouse	15-Jul-2019	57:02	1	6.08	Trauma	LL/BK	R	6	7
002	61	Male	White	College	Divorced	Roommate	13-Nov-2019	27:23	1	7.75	Diabetes	LL/BK	L	7	6
							4-Dec-2019	50:53							
003	52	Male	White	College	Married	Spouse	6-Dec-2019	42:19	1	1.42	Trauma	LL/AK	L	0	1
004	40	Male	White	High School	Married	Spouse	9-Dec-2019	26:23	1	12	Trauma	LL/BK	L	1	1
005	67	Male	White	Bachelor's	Separated	Relatives	17-Feb-2020	53:00	1	1.33	Diabetes	LL/BK	L	3	8
006	65	Male	White	College	Married	Spouse	13-Apr-2022	58:16	1	2.33	Diabetes	LL/BK	R	8	8
007	43	Male	White	Less than High School	Single	Relatives	18-Jul-2023	51:28	1	3.92	Trauma	LL/BK	R	8	8
008	37	Male	East Asian	College	Single	Relatives	21-Jun-2023	39:41	1	5.33	Infection	LL/BK	R	0	0
009	35	Male	White	College	Married	Spouse	15-Sep-2023	39:42	1	4.58	Trauma	LL/AK	R	5	5
010	47	Male	White	Graduate	Married	Spouse	29-Sep-2023	34:23	1	0.92	Trauma	UL/AE	L	7	7
011	67	Female	White	Bachelor's	Widowed	Alone	19-Sept-2023	38:43	1	0.5	Cancer	LL/AK	R	10	8
012	38	Male	South Asian	Bachelor's	In a relationship	Relatives	16-Dec-2024	47:26	4	0.58	Sepsis	UL/BE	R	0	0
												LL/BK	R		
												UL/W	L		
												LL/BK	L		

RLP, residual limb pain; PLP, phantom limb pain; NRS, 0–10 numeric rating scale (0 = no pain, 10 = worst pain imaginable); Min, minutes; Sec, seconds; LL, lower limb; UL, upper limb; BK, below knee; AK, above knee; AE, above elbow; BE, below elbow; W, wrist; R, right; L, left.

**Figure 1:**
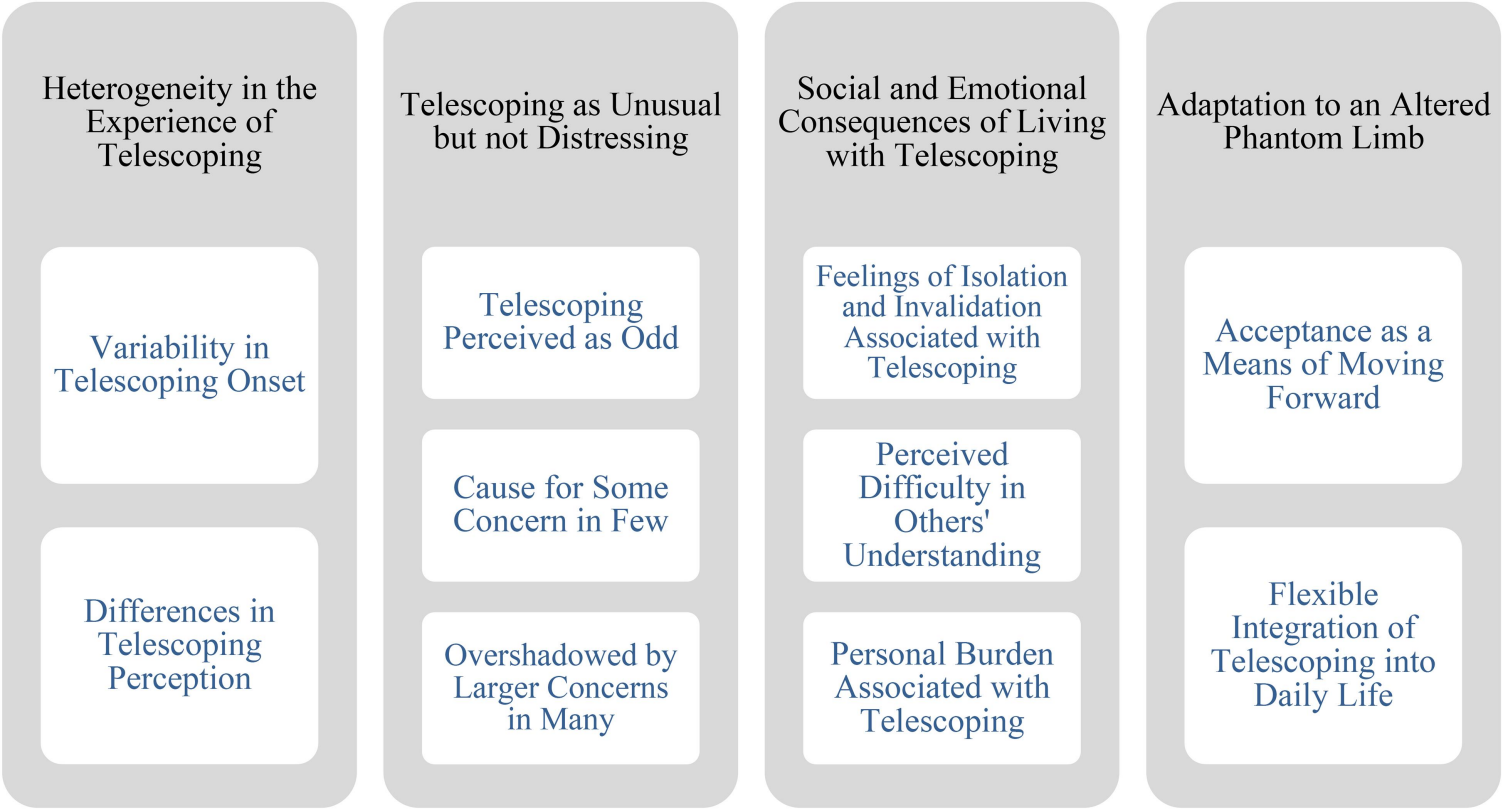
The four main themes and their subthemes.

The inductive thematic analysis resulted in a total of 56 codes, which were subsequently organized into themes and subthemes. Four main themes and 10 subthemes were developed to characterize individuals' experience of phantom limb telescoping (Figure 1). Despite individual differences, every participant's experience of telescoping included at least one element of the following four main categories: (1) *Heterogeneity in the Experience of Telescoping*, in which participants described phantom limb telescoping not as a uniform or predictable process, but as one that was both uniquely experienced and unfolded across time in different ways; (2) *Telescoping as Unusual but Not Distressing*, where participants described telescoping as an unusual sensation which gave rise to potential concerns and/or was overshadowed by larger concerns; (3) *Social and Emotional Consequences of Living with Telescoping*, which represents how participants felt isolated and invalidated as they grappled with their new reality after amputation; and (4) *Adaptation to an Altered Phantom Limb*, which describes the ways participants adapted to the experience of living with an amputated limb(s) and the experience of telescoping. The following sections report the findings using participant quotations to illustrate the subthemes within each main theme. Quotations have been labelled in brackets with the individual's unique ID number and age.

### 3.1 Heterogeneity in the experience of telescoping

#### 3.1.1 Variability in telescoping onset

Participants differed widely in when they first became aware of their phantom limb telescoping. Some perceived it immediately post-amputation while others only experienced it months or years later. Two individuals reported recognition of immediate limb shortening. One participant explained that he experienced a shorter phantom limb “right from the beginning” (P003, 52), and another explained she felt it “right away” (P011, 67), suggesting that for some, telescoping occurs immediately after amputation. Three others first noticed telescoping in the early rehabilitation phase, 4–8 months postamputation, often in conjunction with increased proprioceptive demands of prosthetic use. One participant explained “Six months after the amputation was done when I started consistently wearing a prosthetic leg” (P009, 35) was when he noticed shortening of his phantom limb. This participant was the only one to also spontaneously report a concurrent decrease in his PLP intensity when he experienced his phantom limb telescoping. A third cluster of participants did not become aware of telescoping until one to two years after amputation (*n* = 4). Finally, one participant reported only noticing that his limb was telescoped 12 years after amputation while completing the questionnaires pertaining to this research project. He stated: “It was when I was taking the survey. That was one of the questions on there and I thought about it and then it really does seem like it is shorter than it used to be” (P004, 40). The remaining participants (*n* = 2) could not recall when they had first noticed their limb had first felt shorter than its usual length.

#### 3.1.2 Differences in telescoping perception

Even though each individual experienced telescoping, the way it was perceived varied greatly. Participants described the sensation with reference to personal anatomy, spatial positioning, and metaphors of shrinkage or displacement. Participants explained: “My foot is about six inches higher than it should be from the ground” (P001, 59), “It feels like my foot is in my upper thigh.” (P009, 35), and “My forearm I feel like is no longer there. I do feel like my phantom hand would be very close to where my elbow used to be” (P010, 47). For some, the phantom limb was described as shorter: **“**It just seems to be an inch shorter than it used to be” (P005, 67) and “It actually feels like my hands are really short all of a sudden, […] it feels like I have little arms” (P012, 38). In some cases, prosthetic use altered the experience of the phantom limb. As one participant described, his phantom would sometimes reside in his upper thigh, or change position when using his prosthesis:

Now it's sitting in my prosthetic. So, it's like my phantom is a bit shortened […] I don't [usually] feel the phantom limb anymore. All I feel is the feeling of the prosthetic. And so, I think that […] supersedes maybe the phantom sometimes. (P009, 35)

One participant even described the ability to evoke the sensation through thought, stating: “When I start to think about it, I could make myself feel it” (P009, 35), further emphasizing the flexible nature of phantom limb telescoping awareness.

### 3.2 Telescoping as unusual but not distressing

#### 3.2.1 Telescoping perceived as Odd

Participants commonly described telescoping as an unusual or surreal sensation. Many (*n* = 10) used language such as “odd,” “weird,” or “interesting” to characterize their impressions. One participant exclaimed “That's weird!” (P001, 59), while describing his first reactions to experiencing telescoping. Another participant described it as a “weirder phantom sensation” (P006, 65). Others used non-judgmental, humorous framing, without distress, to highlight how telescoping is perceptually odd, but integrated into their post-amputation experience. For example:

I just assumed it was an oddity in my body or in the way the amputation was done, with the way my body has reacted or the way my brain has, you know, perceived what's happened. Who knows? […] I just chalked it up to the fact that this is neither a right nor wrong, it is simply a symptom of my circumstances. (P003, 52)

Overall, the telescoping experience was not seen as something to be repaired or feared, but rather as a natural extension of an altered phantom limb. Participants highlighted that it was not a sensation that particularly occupied their thoughts. As one participant remarked, the sensation did not elicit any fearful emotions, “I'd say it was interesting. […] I mean I wasn't frightened or anything by it, I just don't know, it's kind of odd” *(*P004, 40). Another individual expanded on this idea further: “It's a very interesting sensation. It's like if somebody cut your forearm out and re-attached […] your hand above [your] elbow. [….] It's not something that really occupies my thoughts” (P010, 47).

#### 3.2.2 Cause for some concern in few

While most participants framed telescoping as a curious or benign oddity, others (*n* = 2) viewed it through more cautious or evaluative lenses. They associated telescoping sensations with uncertainty about prosthetic use, broader post-amputation adaptation outcomes, or the management of PLP. These responses still acknowledged the strangeness of telescoping but layered it with concerns about future impact or overall comfort. One participant shared several of his concerns about the implications of experiencing telescoping in terms of how it may affect his function and recovery:

I […] worry. Just because I'm like "oh, [what] does that affect going forward?" […] I don't know if that means my arms getting weaker or if means it's getting stronger. […] Does that affect if I get prosthetics? Is it always going to have this weird feeling? (P012, 38)

This quote highlights how the sensation itself is not cause for alarm, but its clinical implications are of concern, particularly for prosthetic use or muscle strength. These reactions still fit within the “odd but not alarming” framing, but with a more forward-looking concern. This concern also brings to light the worries of those who experience other non-painful phantom phenomena.

#### 3.2.3 Overshadowed by larger concerns in many

Other participants (*n* = 5) explained that their focus was more on adjusting to life after having lost a limb than making sense of individual sensations. They did not deny or dismiss the sensation but explained how it simply did not register as meaningful compared to the larger psychological and functional adjustments to limb loss they were experiencing. One participant illustrated this concept by explaining, “I wasn't bothered too much by having the feeling up there. It was more so just like, […] please make it comfortable.” (P009, 35). Participants minimized the relevance of telescoping, not because it was not strange, but because PLP was the dominant concern. They prioritized comfort over clarity of sensation, which again reflects that weirdness alone does not predict distress*.* In this context, several participants (*n* = 4) described their awareness of telescoping as minimal or passive, with the phenomenon often going unnoticed or unexamined. For many, telescoping was not a source of distress, curiosity, or focus; it simply existed in the background. Participants shared, “I just never, never thought about it” (P004, 40), “It doesn't really enter my mind a lot.” (P008, 37), and “I noticed it, but didn't pay that much attention to it” (P012, 38). These participants reiterated how their focus was often placed elsewhere, on potentially more distressing or threatening amputation-related experiences and sensations.

### 3.3 Social and emotional consequences of living with telescoping

#### 3.3.1 Feelings of isolation and invalidation associated with telescoping

Participants (*n* = 5) commonly described a sense that their painful and non-painful phantom sensations like telescoping were not taken seriously by others, including healthcare providers, peers, and even themselves. This invalidation arose from interactions with others and their responses to participants' experiences, contributing to feelings of being misunderstood or alone in their experience. A sense of emotional distance from others was a recurring thread. As one individual put it, “I've found other people's reaction is, I feel like they try to understand, but they really don't. Only so far that people can understand without having experienced it” (P009, 35). This lack of understanding sometimes turned into outright dismissal. One participant recalled being told by a chiropodist that their phantom sensations were imagined: “Like, not a real sensation. That [it's] kind of made-up or invented or whatever. And I said, ‘I assure you, it's a very real thing’” (P011, 67)*.* The most acute form of invalidation came from medical professionals. One participant explained:

“It's not recognized as if you were having chronic kidney stones, broken leg, you needed a hip replacement. […] I had one doctor [who] told me it was all in my head. [In a] very insulting way. I was—believe me—insulted.” (P002, 61).

Others described efforts to normalize or rationalize what was happening by assuming it must be common, even in the absence of validation. For example, one participant shared, “I did some research into it, and I just felt that, okay, I guess that's the normal progression of the injury. It happens to other people, so it must be normal” (P010, 47). This strategy appeared to provide temporary reassurance to an individual hoping to cope with the sensation alone but often did not resolve the underlying sense of ambiguity or marginalization.

#### 3.3.2 Perceived difficulty in others' understanding

Despite their vivid and sometimes peculiar experiences associated with telescoping, participants rarely discussed it with others. Many (*n* = 7) described concerns about believability or simply not having the right opportunity or language to share their experiences with others. The sensations were seen as private, strange, or not socially discussable. One participant shared, “I never really talked about it until you brought it up. […] I just—I don't know who you sit around and talk about your phantom limb sensations with. […] I thought it was a situation unique to me” (P003, 52). One individual described how their silence was shaped by fear of being disbelieved or judged:

They'll just think I'm crazy. The pain I can explain to them. They seem to grasp that. Though I'd like to dip their feet in boiling water, so they really experience it. But the telescoping I haven't really mentioned to anybody. (P005, 67)

Pain, while also difficult to communicate, felt more “acceptable” than the bizarre and less visible distortions experienced with a telescoped phantom.

Multiple participants (*n* = 4) noted that they had never brought up the experience to anyone, including medical professionals. Individuals stated: “Have I spoken to anybody? No, not really. […] My amputee doctor hasn't really asked me about it” (P008, 37) and “I may have mentioned it to my team. I can't remember. […] I never specifically addressed a question to any of my medical team about it” (P010, 47). These quotes highlight how many individuals never thought to speak to their medical team about their phantom sensations, nor were they asked about it. Others expressed a willingness to share but noted they simply did not have the opportunity or had not thought to share their phantom sensations with others. One participant reflected:

I'm very open with everything. So, I talk to people about stuff. But like I probably—I don't think I ever brought the feeling of [telescoping] up to anybody. […] Mostly I'll just tell my family about […] the basics when […] I was recovering. But other than that, like, unless it hits that moment I [won't]. (P007, 43)

Similarly, some participants (*n* = 3) admitted to a single, brief conversations with their partner. One individual had described his experience with his wife and stated: “She may be under the impression that that was just a temporary sensation, ‘cause I only think I discussed it with her once” (P003, 52). On the other hand, two participants did talk about their telescoping sensations, typically with those who could personally relate or with whom they had a strong sense of mutual understanding. For example, “I talked to my wife about it. We're both amputees, so we talk about that stuff all the time” (P004, 40). In rare cases, participants found partial recognition from others: “There were some people who got the gist of what I was describing” (P001, 59). Yet, across stories, the sensation was consistently marked as strange, even when shared with someone they felt comfortable with.

#### 3.3.3 Personal burden associated with telescoping

Participants (*n* = 4) commonly explained the way phantom sensations, including telescoping, function as unrelenting reminders of having lost a limb. Participants described how the sensation of having a shorter limb disrupted moments of peace, reasserted the reality of limb loss, and carried emotional weight that challenged efforts to forget, move on, or feel whole again. One participant described a cyclical tug-of-war between forgetting and being pulled back into awareness:

It takes you away from everything. It's almost like when you can finally check out and not really pay attention to it, it brings you back to realizing like "oh wait, [I] lost some limbs." […] It's not very easy to forget because [I] obviously look down and see it all the time. [But when I] stop paying attention to it, it kind of just brings [me] back in. (P012, 38)

Even moments of stillness, like watching TV, could be interrupted by phantom limb telescoping, serving as an involuntary reminder of having lost a limb. This was echoed powerfully by another participant who named the deep internal loss that underpins such reminders: “In that dark, dark space we all have, we're not whole anymore. That plays on [my] mental attitude. Any additional [phantom sensations] are just reminders that [I'm] not” (P006, 65). This blunt reflection highlights how phantom sensations, including telescoping, do not merely disrupt comfort; they actively reinforce a perceived lack of wholeness. While the sensations are not interpreted as inherently harmful or painful, they serve as reminders of what their body has had to endure.

### 3.4 Adaptation to an Altered Phantom Limb

#### 3.4.1 Acceptance as a means of moving forward

Participants (*n* = 6) described how they adapted to the experience of living with phantom limb telescoping. While the sensation was often described as strange, many individuals ultimately integrated telescoping into a new understanding of their bodies, relationships, and everyday lives. This adaptation was neither framed as easy nor complete, but rather as an ongoing process of acceptance, redefinition, and resilience. Multiple individuals (*n* = 4) described a psychological shift toward acceptance of phantom sensations and body changes, often through self-awareness and a focus on regaining function. Phantom sensations became a regular, though strange, part of daily life. One participant explained: “It's become a regular […] condition I live with. And I'm probably like, still on the verge of saying, maybe like, comfortable with at this point. But I can proceed with like, my daily activities” (P009, 35). For others, acceptance was rooted in intentional reframing. One participant described a conscious decision not to let the experience of limb loss or phantom sensations like telescoping define him:

“I made a choice some time ago that […] I wouldn't be defined by what has happened. And then I wouldn't be defined by this experience. Only that I would let it add to who I am. I wouldn't let it take anything away from who I am. […] I think maybe I'm a bit stronger as a result. Maybe a bit tougher mentally as result. But I don't feel like it has taken anything away from who I am.” (P003, 52)

This was echoed by another participant who stated: “I don't let it bother me. If that makes sense. If it's a kind of know something in the back of my mind but it's not really enough to change anything” (P004, 40). This process of individual acceptance reflects a subtle form of resilience, one that does not require the absence of discomfort, but rather an ability to live alongside it.

#### 3.4.2 Flexible integration of telescoping into daily life

Beyond individual acceptance, multiple participants (*n* = 5) described how they had functionally integrated telescoping into their lives in ways that minimized disruption. Participants described flexibility toward their altered phantom limb without being defined or overly limited by it. One participant described how he approached his sensations of telescoping with curiosity rather than fear: “If something happens, I'll be like, well, it's pretty neat. I don't dread it or anything.” (P004, 40). Even those who described the sensations as strange generally did not see them as impairing. As one participant noted*,* “The telescoping and the shrinking of the limb doesn't bother me in the least. It's an odd sensation. It's not going to change my ability to do anything.” (P009, 35). Moreover, most participants (*n* = 8) explained that painful and non-painful phantom sensations, including telescoping, had minimal effects on their daily life or relationships. One participant reflected, “The phantom sensations? No, not really. [The phantom sensations] don't really have an impact.” (P008, 37). Others noted that any effects on relationships were temporary or minor. One person explained that close family and friends had no issue with the phantom limb sensations he experienced, “Once they get past the big shocker, right?” (P006, 65), implying that initial surprise of losing a limb had a greater potential impact on relationships than did telescoping.

## 4 Discussion

The present study aimed to better understand phantom limb telescoping by exploring the phenomenon from the perspective of individuals who experience it. Despite its possible relationship to post-amputation pain, telescoping remains relatively understudied, especially from a qualitative research lens. As such, 12 individuals were included in this study to what the lived experience of phantom limb telescoping is among people who have undergone limb amputation, how it is interpreted, the emotional responses it evoked, and its impact on post-amputation adaptation.

Overall, participants described telescoping as a heterogenous phenomenon that varied in onset, presentation, and personal meaning. Although telescoping was commonly perceived as unusual or strange, it was generally not experienced as inherently distressing and was often overshadowed by broader challenges associated with limb loss. At the same time, telescoping carried social and emotional significance for some individuals, contributing to feelings of invalidation, isolation, and reminders of limb loss. Despite these challenges, participants described adapting to telescoping through the process of acceptance and flexible integration into everyday life.

The current qualitative analysis identified four main themes and ten subthemes, which reflect the multifaceted nature of telescoping. The first theme, *Heterogeneity in the Experience of Telescoping*, comprised two subthemes: *Variability in Telescoping Onset and Differences in Telescoping Perception.* Together, these subthemes illustrate how the experience of telescoping differs from person to person, both in regard to when it is first experienced and how it is described. For some, telescoping was experienced shortly after amputation surgery, while for others it was only noticed years later. Both are consistent with prior research documenting variability in telescoping onset, with a shorter phantom limb being first noticed eight days after amputation to years later (11, 29). This suggests that telescoping may be phenomenon that is reported under specific bodily, cognitive, or social conditions, rather than following a predictable postoperative trajectory (2, 6). One participant only became aware of the shortened length of his phantom limb after completing the pre-interview questionnaire. This highlights how rarely the phenomenon is discussed and how easily it can go unnoticed, especially among those who pay little attention to their phantom limb. These findings align with prior research suggesting that phantom sensations, including telescoping, are shaped by attentional processes, environmental factors, and body schema awareness (30, 31). PLP also increases attention, awareness, and ownership over the phantom limb, and may influence telescoping experience (32). Therefore, variability in when telescoping was first noticed and how it is described may fluctuate based on the co-occurrence of pain.

The second theme, *Telescoping as Unusual but Not Distressing*, encompasses three subthemes: *Telescoping Perceived as Odd, Cause for Some Concern in Few,* and *Overshadowed by Larger Concerns in Many*. This theme captures how most participants describe telescoping as an odd sensation that is not distressing. While this relative lack of distress may partially explain why telescoping remains an understudied phenomenon, telescoping is cause for concern for some. The absence of routine conversations about telescoping in healthcare settings appears to contribute to confusion and worry when individuals notice their phantom limb shortening. Although anxiety was not directly assessed in the present study, these observations may contextualize previous findings that individuals who report telescoping have significantly higher symptoms of anxiety in comparison to those with normal length phantoms (22). Future research may benefit from examining whether uncertainty about or lack of understanding of telescoping contributes to increased anxiety. If so, introducing patients to telescoping and normalizing it could reduce unnecessary anxiety and promote acceptance of a broader range of phantom limb experiences (33). Thus, even though telescoping is not inherently distressing, normalizing the experience can decrease worry as individuals adjust to the loss of their limb.

The third theme, *Social and Emotional Consequences of Living with Telescoping*, included three subthemes: *Feelings of Isolation and Invalidation Associated with Telescoping, Perceived Difficulty in Others' Understanding*, and *Personal Burden Associated with Telescoping.* Together, these themes shed light on the social and psychological challenges that can surround the experience of phantom limb telescoping. Form a social perspective, participants described navigating personal relationships and conversations with healthcare providers where telescoping was met with confusion, skepticism, minimization, or outright dismissal, echoing previous research documenting the invalidation of chronic illness and invisible bodily experiences (34–36). Participants often anticipated that others would not believe them or would not understand their experience. In line with studies on invisible illness and pain disclosure (37), participants often debated when, how, and whether to share their experiences, opting for silence. These findings describe the social experience of living with a condition that cannot be easily described, an aspect that has not been previously documented in the context of phantom limb telescoping.

Telescoping also appeared to have an emotional impact on participants. For many, telescoping was a reminder of a lost limb that activated feelings of grief and sadness. Although depressive symptoms were not assessed in the present study, individuals who report telescoping appear to experience significantly higher symptoms of depression in comparison to those with normal phantom length (22). The grief and sadness described by some participants in this study suggest that telescoping may have emotional implications for some individuals, which is an issue that should be explored in future research. Telescoping may reinforce awareness of limb loss, which, over time, negatively impacts mood. Similarly, the results align with prior research investigating the psychological impact of losing a limb, which highlights themes of loss, identity disruption, and ongoing adaptation (38, 39). Considering both the physical and emotional dimensions of telescoping in healthcare settings may therefore be especially important when considering patients' wellbeing.

The final theme, *Adaptation to an Altered Phantom Limb*, consisted of two subthemes: *Acceptance as a Means of Moving Forward* and *Flexible Integration of Telescoping into Daily Life*. Across participants, telescoping was integrated into everyday life without being fully embraced or resisted. Participants described feelings of acceptance towards their shortened phantom limb and ongoing adjustment of identity, priorities, and self-concept. Some participants chose not to openly discuss their phantom sensations like telescoping with others. Their reluctance may have stemmed in part from a perception that others would not understand, and from the relatively minimal impact of these sensations, which reduced their need to share. Regardless of whether they chose to share these experiences, their narratives reflected a notable degree of flexibility, curiosity, and acceptance. The acceptance described aligns with theoretical models of post-traumatic growth or survival in the face of chronic conditions, which emphasize psychological flexibility and meaning making over removing symptoms (40, 41). Moreover, the ongoing adjustment the participants reported can be seen in contemporary models of grief, where loss of a limb and the experience of telescoping could be a dynamic process of adaptation rather than a linear progression through fixed stages (42).

The composition of the sample should also be considered when interpreting these findings. Most participants had trauma- or cancer-related amputations, which is consistent with previous research suggesting that telescoping is more frequently reported by individuals whose amputations were due to trauma or cancer than peripheral vascular disease (7). The prevalence and intensity of PLP were broadly consistent with previous reports, whereas RLP appeared more common and more severe than has been observed in larger limb loss samples (6).

Overall, the current study underscores that not all post-amputation sensations are interpreted as threatening or distressing. Aligned with previous work, there may be variability in how individuals respond to phantom phenomena, and how these responses are shaped by personal meaning-making, support, and psychological resources (43, 44). These findings suggest that the clinical relevance of telescoping lies more in patient support, normalization, and validation than in managing distress. Participants' accounts suggest that having others acknowledge and discuss the sensation may help individuals feel more supported and understood. Allowing for diverse and non-pathologizing interpretations of phantom limb sensations may therefore be a valuable component of post-amputation care and support.

Integrating qualitative insights of this nature is essential to ensuring that empirical knowledge remains anchored in the realities of those it directly impacts. The narratives presented in this study not only deepen our understanding of the perceptual and bodily complexities of limb loss, but also bring attention to the social and psychological dimensions of adapting to altered phantom sensations. Telescoping was revealed to be more than a neurological event; it is a subjective, evolving experience that intersects with identity, support, loss, and adaptation. For some, it is a neutral or even curious phenomenon; for others, it carries emotional weight. These findings underscore the importance of a biopsychosocial framework in understanding phantom limb phenomena, one that moves beyond pain alone and includes a wider array of sensory and affective experiences.

## 5 Limitations

Several limitations of this study should be acknowledged. First, coding and analysis were primarily conducted by one lead researcher. Although this approach allowed for deeper familiarity with the dataset and contextually grounded interpretations (27), it may have increased the influenced the researcher's perspectives on code and theme development. To enhance the reliability of the analysis, the process was complemented by double-coding and team-based reflexive discussions.

Second, while the analysis has been described in detail, the development of codes and themes remains an interpretive process. As with many qualitative studies, decisions regarding grouping and labelling of themes involved subjective judgment, which may limit the reproducibility of the analytic framework. However, it is important to note that not all qualitative work is meant to be or can be reproducible, and that reproducibility should not be the universally accepted criteria for research quality (45).

Third, some interview questions or follow-up prompts may have unintentionally shaped how participants described their telescoping experiences. The semi-structured interviews encouraged open-ended responses, but they could have been influenced by the framing of certain questions by the interviewer. Moreover, the interview guide did not include questions on all aspects of the postamputation experience (e.g., prosthesis use), which limits the extent to which the study can speak to the full range of participants' lived experiences.

Fourth, the participants were asked to recall the onset and progression of telescoping experiences, which may have introduced memory biases. Given that it had been over 6 years for some participants since they had their limb amputated, their recollection of timing and changes in their phantom limb length may not fully capture the actual experience.

Lastly, the sample lacked demographic diversity, as most of the participants were White men. Similarly, these participants were recruited from a specialized rehabilitation center (St. John's Rehab) and had access to post-amputation health services. While saturation was achieved within this sample of 12 individuals, it is possible that certain subtleties were missed, or that additional themes might have been identified with a more diverse sample in terms of ethnicity, gender, access to healthcare resources, and socioeconomic background. Future research should prioritize recruitment strategies that enhance diversity to explore whether the themes identified here hold across different demographic and cultural contexts.

Despite these limitations, the study offers valuable insights into an underexplored phenomenon and provides a foundation for future research to build upon with more diverse samples and complementary methodologies.

## 6 Conclusions and practice implications

To our knowledge, this study is the first to qualitatively examine the experience of phantom limb telescoping in individuals with limb loss. Despite its relatively frequent occurrence, telescoping has received minimal attention in the literature, particularly from approaches that prioritize accounts of lived experience. The aim of this study was to explore the lived experience of phantom limb telescoping among people who have undergone limb amputation, including their interpretations of telescoping, the emotional responses it evoked, and its impact on post-amputation adaptation. By centering the lived experiences of those directly affected, this study contributes novel insights into a phenomenon that is often overlooked in both research and clinical practice.

From a clinical perspective, this study highlights important practice considerations for health care professionals working with individuals with limb loss. First, normalizing telescoping through early patient education may reduce uncertainty or distress when the phenomenon occurs. Providing clear, non-pathologizing explanations of telescoping as a common and varied post-amputation experience can help patients feel validated and better equipped to interpret their sensations. Second, clinicians should consider integrating open-ended discussions into rehabilitation and follow-up care to allow patients to share sensory experiences that may otherwise go unreported, such as telescoping. Doing so may foster trust, reduce feelings of isolation, and help clinicians identify emerging psychological concerns linked to the phantom experience.

Phantom limb telescoping appears to be a heterogenous and non-distressing phenomenon that is frequently overshadowed by broader post-amputation adjustment and pain. Although many individuals do not describe feeling distressed by telescoping, it may still hold emotional significance. It remains understudied and underdiscussed in healthcare settings. Greater clinical awareness, alongside methodologically rigorous future research, is needed to better understand telescoping's mechanisms, clinical relevance, and possible relationship to post-amputation pain.

## Data Availability

The raw data supporting the conclusions of this article will be made available by the authors, without undue reservation.
